# The impact of hemodialysis on mortality risk and cause of death in *Staphylococcus aureus* endocarditis

**DOI:** 10.1186/s12882-018-1016-0

**Published:** 2018-09-03

**Authors:** Mavish S. Chaudry, Gunnar H. Gislason, Anne-Lise Kamper, Marianne Rix, Anders Dahl, Lauge Østergaard, Emil L. Fosbøl, Trine K. Lauridsen, Louise B. Oestergaard, Christian Hassager, Christian Torp-Pedersen, Niels E. Bruun

**Affiliations:** 1 Department of Cardiology, Herlev-Gentofte Hospital University of Copenhagen, Post 635 Kildegårdsvej 28, 2900 Hellerup, Denmark; 2 The National Institute of Public Health, University of Southern Denmark and The Danish Heart Foundation, Copenhagen, Denmark; 3 Department of Nephrology, University Hospital Copenhagen Rigshospitalet, Copenhagen, Denmark; 4 The Heart Centre, University Hospital Copenhagen Rigshospitalet, Copenhagen, Denmark; 5 Department of Cardiology and Clinical Epidemiology, Aalborg University Hospital and Department of Health Science and Technology, Aalborg University, Aalborg, Denmark; 6 Department of Cardiology, University Hospital Copenhagen Rigshospitalet, Copenhagen, Denmark; 7 Clinical Institute, Aalborg University, Aalborg, Denmark

**Keywords:** *Staphylococcus aureus* endocarditis, Hemodialysis, Mortality

## Abstract

**Background:**

The risk of infective endocarditis (IE) is markedly increased in patients receiving chronic hemodialysis compared with the general population, but outcome data are sparse. The present study investigated causes and risk factors of mortality in a hemodialysis-treated end-stage kidney disease- (ESKD) and a non-ESKD population with *staphylococcus* (*S.*) *aureus* endocarditis.

**Methods:**

Hemodialysis-treated ESKD patients with *S. aureus* endocarditis were identified from Danish National Registries and Non-ESKD patients from The East Danish Database on Endocarditis. For establishing the cause of death The Danish Registry of Cause of Death was used. Independent risk factors of outcome were identified in multivariable Cox regression models.

**Results:**

One hundred twenty-one hemodialysis patients and 190 non-ESKD patients with *S. aureus* endocarditis were included during 1996–2012 and 2002–2012, respectively. The all-cause in-hospital mortality was 22.3% in hemodialysis- and 24.7% in non-ESKD patients. One-year mortality, excluding in-hospital mortality, was 26.4% in hemodialysis patients and 15.2% in non-ESKD patients.

The hazard ratio of all-cause mortality in hemodialysis was 2.64 (95% CI 1.70–4.10) at > 70 days after admission compared with non-ESKD. Age (HR 1.03 (95% CI 1.02–1.04)) and diabetes mellitus (HR 2.17 (95% CI 1.54–3.10)) were independent risk factors of all-cause mortality. The hazard ratio of cardiovascular death in hemodialysis was 3.20 (95% CI 1.78–5.77) at > 81 days after admission compared with non-ESKD. Age and diabetes mellitus were independently related to cardiovascular death.

**Conclusion:**

All-cause in-hospital mortality rates were similar in hemodialysis and non-ESKD patients with *S. aureus* endocarditis whereas one-year mortality rates were significantly increased in the hemodialysis population.

**Electronic supplementary material:**

The online version of this article (10.1186/s12882-018-1016-0) contains supplementary material, which is available to authorized users.

## Background

The risk of infective endocarditis (IE) is high in patients with end-stage kidney disease (ESKD) [1]. Overall, the most common microbial cause of IE in Denmark is streptococcal species [2]. However, hemodialysis is associated with frequent *staphylococcus* (*S.*) *aureus* bacteremia. The risk factors include repeated access to the vascular system required for hemodialysis, a high frequency of underlying heart valve disease and uremia related immune impairment [3–7]. The all-cause in-hospital mortality has been reported as high as 52% in hemodialysis patients with IE and up to 56.3% at one-year follow-up [8–11]. Mitral valve disease and septic embolism have been identified as mortality risk factors in hemodialysis patients with IE [12].

The all-cause in-hospital mortality of *S. aureus* endocarditis in the general population ranges between 15 and 22%, depending on the geographic region and study population [13–15]. Heart failure, age and cerebrovascular events are reported as mortality risk factors in the general IE population [13, 15].

However, it remains to be clarified whether outcomes, patient characteristics, cause of death and mortality risk factors differ between hemodialysis- and non-ESKD *S. aureus* endocarditis patients.

Therefore, the current study aimed to compare mortality, causes of death, and independent mortality risk factors in hemodialysis-treated ESKD patients and non-ESKD patients with *S. aureus* endocarditis.

## Methods

In Denmark, all residents are provided with a permanent personal identification number that allows linkage between nationwide administrative registries on an individual level. Four of these registries were utilized to obtain data. The Danish National Patient Registry includes information on all outpatient appointments and hospital admissions including diagnoses and procedural codes in Denmark, since 1978. Each admission and outpatient appointment is at discharge and at end of consultation coded with one primary diagnosis, and if appropriate one or more secondary diagnoses, according to the International Classification of Diseases - until 1994 the 8th revision (ICD-8) was used and from 1994 the 10th revision (ICD-10) has been applied [16]. The codes used to retrieve comorbidities are considered valid [17]. All deaths in Denmark are registered in The National Civil Registry within 2 weeks from time of death. The Danish Registry of Cause of Death holds information on cause of death among deceased Danish residents in Denmark on an individual level since 1970 and is coded according to ICD-10, since 1994 [18]. The Danish National Registry on Regular Dialysis and Transplantation was established in 1990 and contains data on all Danish patients receiving renal replacement therapy, including changes in treatment modality and is considered valid [19].

Data in The East Danish Database on Endocarditis was prospectively collected with consecutive enrolment of patients diagnosed with IE from October 1st, 2002 to December 31st, 2012, at two tertiary referral heart centers in Copenhagen, Denmark. The centers cover a catchment area of more than 2.4 million people. The diagnosis of IE was based on clinical, microbiological and echocardiographic findings evaluated according to the revised Duke criteria [20]. The population has been described in more details previously [21].

### Population

The study comprised a hemodialysis population and a non-ESKD population with *S. aureus* endocarditis.

The hemodialysis population was identified from The Danish National Registry on Regular Dialysis and Transplantation in the period from January 1st, 1996 to December 31st, 2012.

Each hemodialysis patient was included at the first episode of IE after initiation of renal replacement therapy, if caused by *S. aureus*. Patients were identified according to hospital admission with discharge ICD-10 codes I33 and I38 as recorded in The Danish National Patient Registry. The codes are considered accurate and valid [22, 23]. Information on microbiology was based on blood cultures retrieved from all Danish Departments of Microbiology. Medical records were reviewed to collect information on echocardiography and the involved heart valves in each individual.

The non-ESKD population was identified from The East Danish Database on Endocarditis. Patients with acute kidney failure requiring temporary hemodialysis treatment during admission were included in the cohort. Patients in renal replacement therapy (hemodialysis, peritoneal dialysis and kidney transplanted) were excluded from the non-ESKD population.

### Comorbidity

Data on comorbidity was derived from The Danish National Patient Registry in a period of five years before index.

### Outcome

The outcomes of interest were all-cause mortality as well as mortality subdivided into cardiovascular- and non-cardiovascular mortality. Cardiovascular death was considered present when at least one diagnosis on the death certificate was cardiovascular (I-diagnosis by ICD-10, Additional file 1: Table S3). All subjects were followed until death or end of study, December 31st, 2012.

### Ethics

The study was approved by the Danish Data Protection Agency (ref. 2007–58-0015 / internal ref. GEH-2014-015 I-suite no. 02733). Retrospective studies in registries do not require ethical approval in Denmark.

### Statistical analyses

Continuous variables were represented as mean +/− standard deviation. Chi-square- and Fischer’s exact test were used for analyses of differences between categorical variables. Rank sum tests were used for differences between continuous variables. Cox proportional hazard models were used to examine time from endocarditis to death or study end. The following covariates were included in the models: sex, age, diabetes mellitus and exposure (hemodialysis, non-ESKD). The hazard ratio between hemodialysis and non-ESKD changed (violation of proportional hazard assumption) over time and was therefore examined in discrete time periods of below 20 days, 20–70 days and at least 71 days for the outcome, all-cause mortality, and in time periods of below 26 days, 26–81 days and at least 82 days for the outcome, cardiovascular death. These periods were selected to represent the first- and second quartile of outcome. Cumulative incidence curves were depicted for all-cause mortality and for the following end-points: cardiovascular death and non-cardiovascular death accounting for competing risks of death.

Sensitivity analyses were performed, excluding hemodialysis patients switching renal replacement therapy modality during the study period, to validate the findings of multivariable Cox regression and cumulative incidence.

All statistical analyses were performed using SAS version 9.4 (SAS institute, Cary, NC, USA).

## Results

### Characteristics of the study population

Among a total number of 8791 hemodialysis patients, 121 patients with *S. aureus* endocarditis were identified (Additional file 1: Tables S1 and S2). In non-ESKD patients, 190 patients with *S. aureus* endocarditis were included from the 977 IE patients in the East Danish database on Endocarditis. Of these, 31 patients experienced acute kidney failure during admission and received temporary hemodialysis treatment. Previous endocarditis was identified in 10 hemodialysis patients and 10 non-ESKD patients. The mean age was 60.5 (+/-SD 15.2 years) in the hemodialysis population and 62.3 (+/-SD 16.2 years) in the non-ESKD population.

More males than females were hospitalized with *S. aureus* endocarditis during the study period (*p* < 0.001).

Diabetes mellitus and peripheral vascular disease were significantly more prevalent in hemodialysis- than in non-ESKD patients. Baseline characteristics and distributions of comorbidities are presented in Table 1 (Additional file 1: Table S3).

**Table 1:** Baseline characteristics of study populations with *Staphylococcus aureus* endocarditis

Characteristics	Hemodialysis (*n* = 121)	Non-ESKD (*n* = 190)	Total (*n* = 311)	*P* value
Follow-up (years)	1.8 ± 2.4	2.6 ± 2.9	2.3 ± 2.8	
Age (years)	60.5 ± 15.2	62.3 ± 16.2	61.6 ± 15.8	0.301
Female (N)	47 (39%)	52 (27.4%)	99 (31.8%)	0.045
Age (years)	57.9 ± 14.7	66.6 ± 17.4	62.5 ± 16.5	0.004
Male (N)	74 (61%)	138 (72.6%)	212 (68.2%)	0.045
Age (years)	62.1 ± 15.3	60.6 ± 15.6	61.2 ± 15.5	0.417
Pre-existing Heart valve disease				
Aortic valve	17 (14.1%)	40 (21.1%)	57 (18.3%)	0.134
Mitral valve	12 (10%)	22 (11.6%)	34 (11%)	0.712
Comorbidity				
Myocardial infarction	17 (14.1%)	22 (11.6%)	39 (12.5%)	0.598
Diabetes mellitus	43 (35.5%)	23 (12.1%)	66 (21.2%)	< 0.001
Chronic obstructive lung disease	7 (5.8%)	7 (3.7%)	14 (4.5%)	0.410
Peripheral vascular disease	19 (15.7%)	12 (6.3%)	31 (10%)	0.012
Ischemic heart disease	32 (26.5%)	48 (25.3%)	80 (25.7%)	0.894
Cardiac arrythmia disorder	30 (24.8%)	36 (19%)	66 (21.2%)	0.255
Atrial flutter	25 (20.7%)	26 (13.7%)	51 (16.4%)	0.118
Chronic heart failure	30 (24.8%)	32 (16.8%)	62 (20%)	0.109
^a^Chronic kidney disease stage				
1		51 (26.8%)		
2		52 (27.4%)		
3		55 (28.9%)		
4		25 (13.2%)		
5		7 (3.7%)		
5 dialysis	121 (100%)	–		

Values are given as mean, +/− SD or N (%)

^a^The plasma creatinine level at admission was used to calculate the eGFR and the CKD-EPI creatinine equation was applied

### Mortality, cause of death and heart valve surgery

During the study period, all-cause mortality was higher in hemodialysis- (80.2%) than in non-ESKD patients (56.8%), (*p* < 0.001). Cardiovascular death was also higher in the hemodialysis- (46.3%) than in the non-ESKD population (32.1%), (*p* = 0.016). The mean follow-up was 2.3 (+/-SD 2.8 years) in the study population.

The all-cause in-hospital mortality was similar, 22.3% and 24.7% in hemodialysis- and non-ESKD patients, respectively. The all-cause one-year mortality, excluding in-hospital mortality, was higher in hemodialysis- than in non-ESKD patients (*p* = 0.023), Table 2.

**Table 2:** In-hospital- and one-year mortality following *Staphylococcus aureus* endocarditis

	In-hospital			1-year excluding in-hospital		
Cause of Death	Hemodialysis (*n* = 121)	Non-ESKD (*n* = 190)	*P* value	Hemodialysis (*n* = 121)	Non-ESKD (*n* = 190)	*P* value
All-cause	27 (22.3%)	47 (24.7%)	0.683	32 (26.4%)	29 (15.3%)	0.023
Cardiovascular	14 (11.6%)	26 (13.7%)	0.729	18 (14.9%)	17 (8.9%)	0.123
Heart failure	2 (1.7%)	7 (3.7%)		4 (3.3%)	10 (5.3%)	
Myocardial infarction	–	2 (1.1%)		2 (1.7%)	1 (0.5%)	
Stroke	3 (2.5%)	4 (2.1%)		4 (3.3%)	4 (2.1%)	
^a^Other	9 (7.4%)	5 (2.6%)		13 (10.7%)	3 (1.6%)	
Non-cardiovascular	13 (10.7%)	21 (11.1%)	0.932	11 (9.1%)	11 (5.8%)	0.298
Sepsis	10 (8.3%)	14 (7.4%)		5 (4.1%)	3 (1.6%)	
Respiratory failure	3 (2.5%)	2 (1.1%)		3 (2.5%)	1 (0.5%)	
ESKD	–	–		2 (1.7%)	–	
Diabetes mellitus	–	–		2 (1.7%)	1 (0.5%)	
Gangrene	–	–		–	1 (0.5%)	
Unknown	–	5 (2.6%)		–	5 (2.6%)	

Values are given as N (%), − None

^a^Aortic valve disease, mitral valve insufficiency, ventricular fibrillation, vascular hypertension, cardiac arrest, atrial fibrillation, unclassified cardiovascular cause of death subsequent to *Staphylococcus aureus* endocarditis

‡ESKD end-stage kidney disease

The mitral valve was most often affected in the hemodialysis population, whereas infections of the mitral- and aortic valves were more evenly distributed in the non-ESKD patients, Table 3. All patients underwent echocardiography. The proportion of transesophageal echocardiography (TEE) was 70% in the hemodialysis population and 92.6% in the non-ESKD population.

**Table 3:** Heart valve involvement in *Staphylococcus aureus* endocarditis

	Hemodialysis (*n* = 121)	Non-ESKD (*n* = 190)	Total (*n* = 311)
Mitral valve	62 (51.2%)	50 (26.3%)	112 (36%)
Aortic valve	27 (22.3%)	72 (37.9%)	99 (31.8%)
Tricuspid valve	3 (2.5%)	13 (6.8%)	16 (5.1%)
Pulmonic valve	–	1 (0.5%)	1 (0.3%)
Aortic- and mitral valve	3 (2.5%)	25 (13.2%)	28 (9%)
Aortic- and tricuspid valve	–	1 (0.5%)	1 (0.3%)
Aortic- and pulmonic valve	–	1 (0.5%)	1 (0.3%)
Mitral- and tricuspid valve	3 (2.5%)	1 (0.5%)	4 (1.3%)
Mitral- and pulmonic valve	–	2 (1.1%)	2 (0.6%)
Cardiac device lead	5 (4.1%)	22 (11.6%)	27 (8.7%)
Right side	9 (7.4%)	2 (1.1%)	11 (3.5%)
Unavailable	9 (7.4%)	–	9 (2.9%)

Values are given as N (%), − None

Information on echocardiography was not available in nine hemodialysis patients.

A larger proportion of non-ESKD- than hemodialysis patients underwent heart valve surgery during admission. The one-year mortality subsequent to surgical treatment was higher in the hemodialysis population than in the non-ESKD population, Table 4. In the non-surgically treated patients, the mortality at one year follow-up was 48.5% in the hemodialysis population and 43.3% in the non-ESKD population. In the non-surgically treated hemodialysis patients the mitral valve was infected in 48.5% and the aortic valve in 22.8%, as compared with 20.1% and 38.1% in the non-ESKD patients, respectively.

**Table 4:** Heart valve surgery and mortality concomitant to *Staphylococcus aureus* endocarditis

	Heart valve surgery								Mortality	
	Total	Mitral valve	Aortic valve	Tricuspid valve	Pulmonic valve	Aortic- and mitral valve	Aortic- and tricuspid valve	Mitral- and tricuspid valve	In-hospital	1-year including in-hospital
Hemodialysis	20	13 (65%)	4 (20%)	–	–	3 (15%)	–	–	4 (20%)	10 (50%)
Non-ESKD	56	23 (41.1%)	21 (37.5%)	3 (5.4%)	2 (3.6%)	5 (9%)	1 (1.8%)	1 (1.8%)	13 (23.2%)	18 (32.1%)

Values are given as N (%)

− None

### Factors associated with mortality

**Fig. 1:**
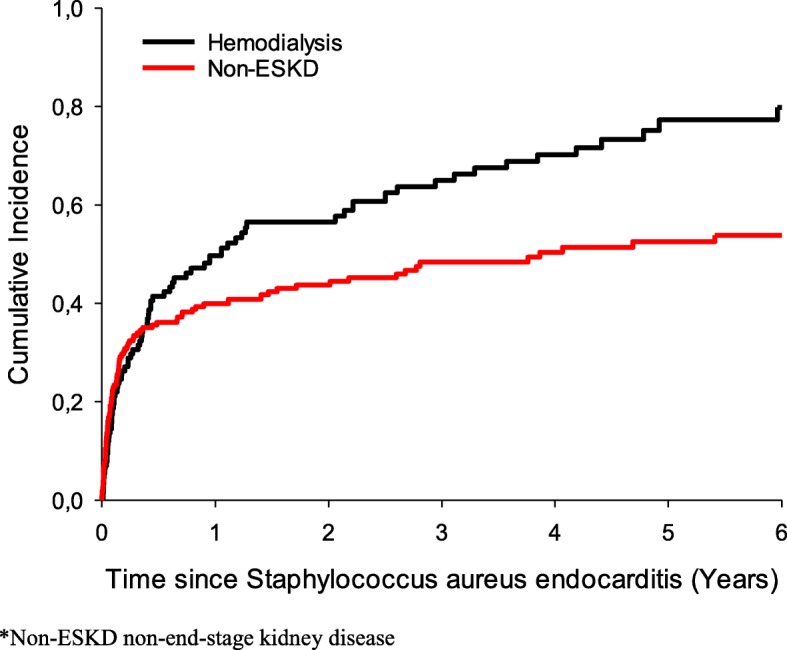
Cumulative incidence of all-cause mortality according to exposure (hemodialysis in ESKD, non-ESKD)

After an initial period of similar all-cause mortality in hemodialysis- and non-ESKD patients, the risk of all-cause mortality in hemodialysis patients exceeded the all-cause mortality risk in the non-ESKD population, Fig. 1.

**Fig. 2:**
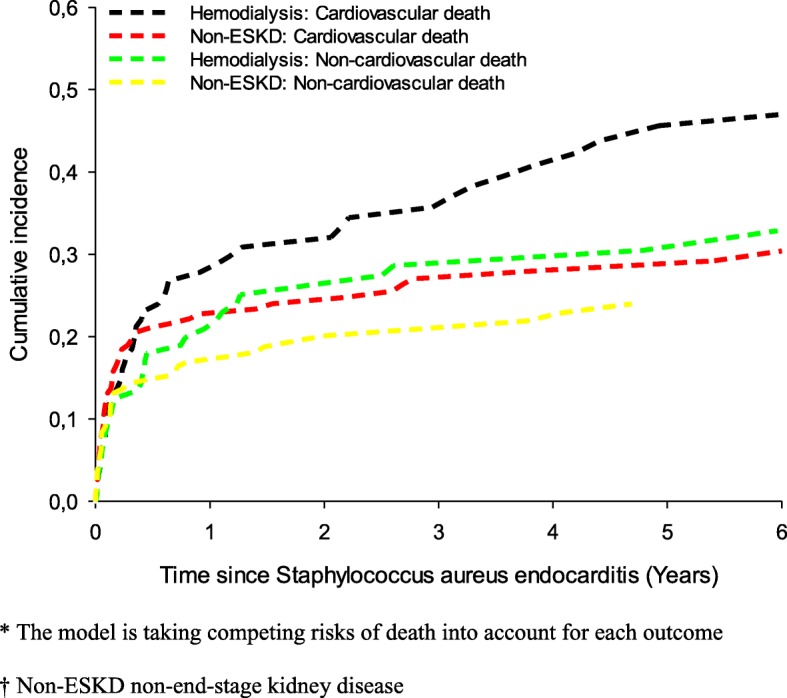
Cumulative incidence of cardiovascular death and non-cardiovascular death according to exposure (hemodialysis in ESKD, non-ESKD)

Similarly, there was no initial difference in the risk of cardiovascular- and non-cardiovascular death in the two study populations, but after an initial period the risk of cardiovascular death in hemodialysis patients exceeded the risk in non-ESKD patients, Fig. 2.

**Fig. 3:**
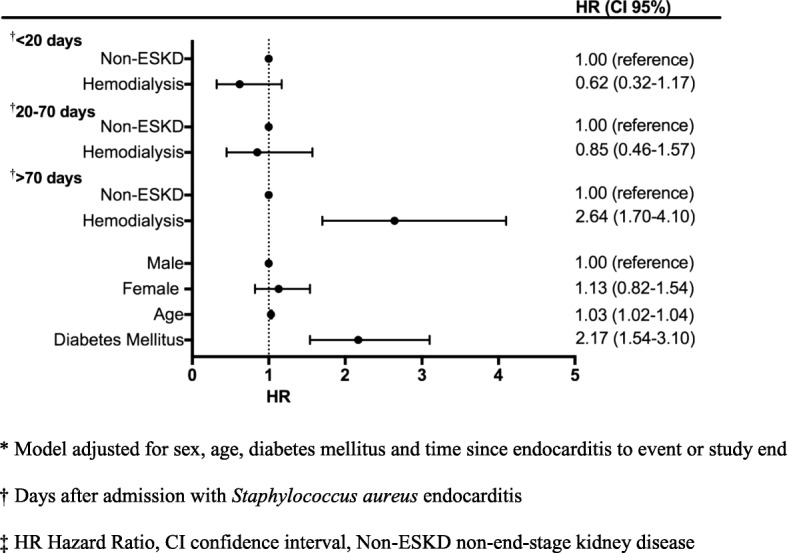
Risk factors for all-cause mortality in *Staphylococcus aureus* endocarditis patients

The hazard ratios of all-cause mortality did not differ between hemodialysis- and non-ESKD patients during the first two time periods: < 20 days- and 20–70 days after admission, but after 70 days, the risk of all-cause mortality in hemodialysis patients increased significantly, HR 2.64 (95% CI 1.70–4.10), compared with non-ESKD patients. Diabetes mellitus and age were independently associated with all-cause mortality. There was no association between gender and all-cause mortality, Fig. 3.

**Fig. 4:**
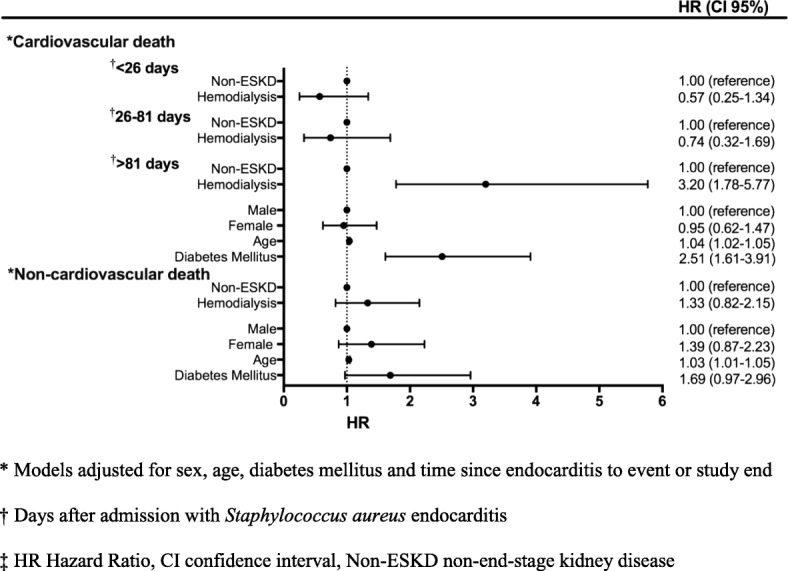
Risk factors for cardiovascular- and non-cardiovascular death in *Staphylococcus aureus* endocarditis patients

Figure 4 shows the results of the adjusted multivariable Cox regression for each end-point of death. The hazard ratios of cardiovascular death were similar in both study populations during the first two time periods: < 26 days- and 26–81 days after admission. At the third time period (> 81 days after admission), the risk of cardiovascular death was increased in hemodialysis patients compared with non-ESKD patients, HR 3.20 (95% CI 1.78–5.77). Age and diabetes mellitus were significantly related to cardiovascular death.

The associations between exposure (hemodialysis compared with non-ESKD), gender, age and non-cardiovascular death were statistically insignificant.

### Sensitivity analyses

Ten hemodialysis patients with *S. aureus* endocarditis changed treatment modality during follow-up to either peritoneal dialysis or received kidney transplantation. Sensitivity analyses were performed excluding these ten patients. The association between exposure (hemodialysis compared with non-ESKD) and adverse end-points remained consistent and the cumulative incidences remained unaltered.

The mortality at one year was 67.7% and 34.6% in the 31 non-ESKD patients with temporary hemodialysis treatment and the remaining 159 non-ESKD patients (*p* = 0.001), respectively. Sensitivity analysis was performed, excluding the 31 non-ESKD patients with temporary hemodialysis treatment. The association between exposure (hemodialysis compared with non-ESKD) and all-cause mortality remained valid.

Sensitivity analysis was performed, excluding 31 patients initiating hemodialysis during 1996–2001. The HR of all-cause mortality in hemodialysis was 0.64 (95% CI 0.34–1.19) < 22 days, 0.83 (95% CI 0.44–1.56) at 22–70 days and 2.64 (95% CI 1.70–4.08) at > 71 days after admission compared with non-ESKD. Thus, the association between the exposure variable and all-cause mortality remained unaltered.

## Discussion

There were two major findings in this study. First, in patients with *S. aureus* endocarditis in-hospital mortality was high, but there was no difference between hemodialysis and non-ESKD patients. Second, in patients discharged alive, mortality remained high in the one-year follow-up period. The one-year mortality in hemodialysis patients with *S. aureus* IE remained at the same level as in-hospital and higher than the annual mortality in the general Danish dialysis population [24, 25]. In non-ESKD patients with *S. aureus* IE, mortality declined by approximately one third at one-year follow-up compared with the in-hospital mortality.

Overall, the distribution of basic characteristics in hemodialysis- and non-ESKD patients was similar, except from the prevalence of diabetes and peripheral vascular disease, which were significantly higher in hemodialysis- compared with non-ESKD patients. These differences in baseline characteristics are common features of hemodialysis patients [1]. It is observed that the women in the hemodialysis population are younger than the women in the non-ESKD population, which remains to be explored further.

The present all-cause in-hospital mortality rate in hemodialysis patients was comparable to findings in other studies on hemodialysis and IE. In a large series of 13,130 hemodialysis patients with IE, Shroff et al. found an in-hospital mortality rate of 23.5% [26]. In smaller observational studies in-hospital- and 30 days mortality were reported to be 14.3–31% [3, 8, 27, 28]. In a recent Danish study, Ludvigsen et al. included 9392 hemodialysis patients, of these 150 patients were diagnosed with IE [29]. The thirty-day mortality was 15% in IE patients undergoing hemodialysis, which is slightly lower than the in-hospital mortality in the present study. The reported thirty-day mortality in the study by Ludvigsen et al. is based on IE regardless of bacterial etiology, which might explain the observed difference.

The in-hospital mortality varied between 15 and 22% in other recently studied populations [13–15], which is in accordance with our current observation. However, patients in renal replacement therapy were not excluded from previous studies.

In the present study, the in-hospital mortality was similar for hemodialysis- and non-ESKD patients. Likewise, Hsiao et al. found evenly distributed in-hospital mortality in 39 hemodialysis patients (46.2%) and 51 non-ESKD patients (51%) with IE [30]. However, these in-hospital mortality rates were markedly higher than those in the present study. This difference might be caused by non-comparable study populations. In our study, 70% of hemodialysis patients underwent TEE. The sensitivity of TEE is superior to transthoracic echocardiography (TTE) not only in detecting perivalvular abscesses, −fistulas and perforations, but also in detecting small vegetations [31, 32]. The outcome of uncomplicated IE patients with small vegetations is often good, and patients with complicated IE who receive surgery have a definite better outcome than complicated IE treated conservatively [33]. In the study by Hsiao et al., the percentage of TEE is unclear and only TTE data is referred. Thus, a lower proportion of TEE may have led to selection bias in disease severity, by including patients with only larger vegetations and unrecognized perivalvular complications, which in part may explain their higher in-hospital mortality rates.

The all-cause one-year mortality in hemodialysis- and non-ESKD patients was similar to other studies on IE. In a single center study of 2239 hemodialysis patients from the period 1990–2000, Maraj et al. identified 24 incident cases of *S. aureus* endocarditis among 32 cases of IE [34]. They found a one-year all-cause mortality of 56.3% among all IE cases. In a study from the International Collaboration on Endocarditis-cohort, Lauridsen et al. reported the all-cause one-year mortality among patients with left-sided native valve *S. aureus* endocarditis to 43%, which is consistent with the current non-ESKD population [35]. Hemodialysis patients were not excluded in the study by Lauridsen et al., but only accounted for 12% of their population.

Patients undergoing dialysis have remarkably high one-year mortality. Cardiovascular disease is recognized as the leading cause of death in dialysis patients [36] followed by infections frequently related to the hemodialysis vascular access [37]. Recurrent bacteremia due to impaired immune function second to uremia [4] and the reduced defense against bacteria following repeated access to the vascular system dispose to increased risk of infections [38]. The all-cause one-year mortality was 16–20% in the Danish dialysis population in the period 2014–2015 [24, 25]. In a recent study on a large European end-stage kidney disease population from 1993 to 2007 with follow-up until 2012, Vogelzang et al. demonstrated an all-cause long-term mortality of 67% in the studied period with a median follow-up of 3.7 years. Cardiovascular death accounted for 24.4%, whereas infections were ascribed as the cause of death in 11% [39]. However, hemodialysis patients with *S. aureus* endocarditis have an even higher one-year cardiovascular mortality and a higher long-term cardiovascular and all-cause mortality.

Left-sided involvement in IE is most common in hemodialysis patients [11, 27, 28]. Calcification of the aortic- and mitral valve is common in end-stage kidney disease, which leads to altered local hemodynamics, increasing the susceptibility for IE [40, 41]. The right sided heart valves were involved in nine cases only in the present hemodialysis population, which is consistent with previous published data [11, 12, 27, 28]. It has been reported that the mitral valve is most commonly involved in left-sided IE in hemodialysis patients [11, 12, 27, 28], which may be due to annular calcification of the mitral valve [42]. In accordance with this observation we found mitral valve endocarditis in 51.2% of the hemodialysis patients.

Heart valve surgery was performed twice as often in the non-ESKD population as in the hemodialysis population. This difference may be explained by the high number of comorbidities leading to increased risk of heart valve surgery in hemodialysis patients. The in-hospital mortality following surgery was similar in both populations, but differed at one-year follow-up. Restriction in selection of hemodialysis patients eligible for surgery may explain the similar in-hospital mortality. In other studies, in-hospital mortality following heart valve surgery in hemodialysis patients has been varying (7–73%) [8, 12, 27, 43]. Differences in patient selection and disease severity at the time of intervention may be part of the explanation.

Raza et al. [44] found a higher mortality at one-year among surgically treated hemodialysis patients with IE compared with a propensity matched group of non-ESKD population and compared with the general hemodialysis population. This is consistent with our data. However, Raza et al. also found that mortality at one-year was higher in the non-surgically treated hemodialysis population (70%) with IE than in the surgically treated hemodialysis population (44%). We found no difference in the one-year mortality in the operated- (50%) and the non-operated (48.5%) hemodialysis patients.

The mortality rate related to mitral valve endocarditis has recently been reported higher than in aortic valve endocarditis. Surgical challenges related to invasive mitral endocarditis compared with aortic valve surgery complicate the surgical treatment and outcome of mitral valve endocarditis [45]. The proportion of mitral valve endocarditis was higher in the non-surgically treated hemodialysis population reported by Raza et al. (62%) compared with the present study (48.5%), which may explain a lower mortality rate in our study.

Additional factors may contribute to this discrepancy. Our study population was smaller and unmatched. Furthermore, possible differences in distribution of methicillin resistance *S. aureus* and geographical area may explain the findings.

In non-ESKD patients, age, heart failure and cerebrovascular event have been described as risk factors of all-cause death in IE [14, 15, 46]. In hemodialysis patients, age, cerebrovascular event and diabetes mellitus have been related to all-cause mortality [12, 26]. In line with these previous data, diabetes mellitus and age were also related to both all-cause- and cardiovascular mortality in the current study. In addition, hemodialysis was associated with increased risk of all-cause mortality and cardiovascular death 70- and 81 days after admission with hazard ratios of 2.64 (95% CI 1.70–4.10) and 3.20 (95% CI 1.78–5.77) compared with non-ESKD patients, respectively. These comparisons have not been reported before and might be explained by a combination of frequent and continuous calcification of heart valves and high prevalence of mitral valve involvement in infective endocarditis, leading to progressive heart failure in the hemodialysis population with IE [40].

### Strengths and limitations

There are limitations inherent to the observational design of the study population. Furthermore, the study population is small and unmatched. However, the hemodialysis cohort is nationwide and includes all chronic hemodialysis patients with *S. aureus* endocarditis in Denmark in the study period, based on a validated nationwide registry. Additionally, each record for hemodialysis patients was assessed to retrieve information on the microbiological agent, echocardiography and infected heart valve. There are some limitations in the non-ESKD cohort. It consists of patients from two tertiary heart centers and is therefore subject to referral bias. Complicated patients and those eligible of surgery are referred to these centers, thus limiting the generalizability of the results. However, it should be noted that the two centers covered a catchment area of > 2.4 million people. Information on methicillin resistant *Staphylococcus aureus* (MRSA) is not available in the national registries. However, the occurrence of MRSA infection is low in Denmark [47].

## Conclusions

In patients with *S. aureus* endocarditis, despite similar in-hospital mortality, chronic hemodialysis treatment had a marked negative impact on one-year all-cause- and cardiovascular mortality as compared with non-ESKD patients. This difference in mortality was significant within 3 months.

## Additional files

Additional file 1: **Table S1.** Renal baseline characteristics of the hemodialysis study population with *Staphylococcus aureus* endocarditis. **Table S2.** ICD-10 codes used to define the etiology of kidney disease of the subset “other”. **Table S3.** International Classification of Diseases 8 and 10 codes used to define comorbidity and outcome in the study population. The supplemental tables contain information on the renal baseline characteristics of the hemodialysis population, diagnosis- and pharmacotherapy codes used to determine comorbidity and origin of kidney disease in the study population. (DOCX 25 kb)

## Abbreviations

ESKD: End-stage kidney disease
HR: Hazard ratio
ICD: International Classification of Diseases
IE: Infective endocarditis
MRSA: Methicillin resistant *Staphylococcus aureus*
*S. aureus*: *Staphylococcus aureus*
TEE: Transesophageal echocardiography
TTE: Transthoracic echocardiography
