# Supplementary material for: The impact of hemodialysis on mortality risk and cause of death in Staphylococcus aureus endocarditis
Source: BMC Nephrol. 2018 Sep 3;19:216. doi: 10.1186/s12882-018-1016-0 (PMC6122200; doi:10.1186/s12882-018-1016-0)
Supplement: Supplementary file 1 — Table S1. Renal baseline characteristics of the hemodialysis study population with Staphylococcus aureus endocarditis. Table S2. ICD-10 codes used to define the etiology of kidney disease of the subset “other”. Table S3. International Classification of Diseases 8 and 10 codes used to define comorbidity and outcome in the study population. The supplemental tables contain information on the renal baseline characteristics of the hemodialysis population, diagnosis- and pharmacotherapy codes used to determine comorbidity and origin of kidney disease in the study population. (DOCX 25 kb) [file 12882_2018_1016_MOESM1_ESM.docx]

**The Impact of Hemodialysis on Mortality Risk and Cause of Death in Staphylococcus aureus Endocarditis**

Mavish S. Chaudry MD^1^, Gunnar H. Gislason MD, PhD^1,2^, Anne-Lise Kamper MD, DMSc^3^, Marianne Rix MD, PhD^3^, Anders Dahl MD^1^, PhD, Lauge Østergaard MB^4^, Emil L. Fosbøl MD, PhD^4^, Trine K. Lauridsen MD, PhD^1^, Louise B. Oestergaard MD^1,5^, Christian Hassager MD, DMSc^6^, Christian Torp-Pedersen MD, DMSc^5^, Niels E. Bruun MD, DMSc^1,7^

1 Department of Cardiology, Herlev-Gentofte Hospital University of Copenhagen, Denmark

2 The National Institute of Public Health, University of Southern Denmark and The Danish Heart Foundation, Copenhagen, Denmark

3 Department of Nephrology, University Hospital Copenhagen Rigshospitalet, Denmark

4 The Heart Centre, University Hospital Copenhagen Rigshospitalet, Denmark

5 Department of Cardiology and Clinical Epidemiology, Aalborg University Hospital and Department of Health Science and Technology, Aalborg University, Aalborg, Denmark

6 Department of Cardiology, University Hospital Copenhagen Rigshospitalet, Denmark

7 Clinical Institute, Aalborg University, Aalborg, Denmark.

Address for correspondence:

Mavish Safdar Chaudry, MD,

Department of Cardiology, Gentofte Hospital, Post 635

Kildegårdsvej 28, 2900 Hellerup, Denmark

E-mail: mavish89@hotmail.com

Phone: (+45)28112786 FAX: (+45)70201281

**Additional file 1: Table S1. Renal baseline characteristics of the hemodialysis study population with *Staphylococcus aureus* endocarditis**

| **Characteristics** | **Hemodialysis**  **(n = 121)** |
| --- | --- |
| **Etiology of kidney disease** |  |
| Diabetes mellitus | 39 (32.2%) |
| Chronic glomerulonephritis | 16 (13.2%) |
| Vascular and hypertensive nephropathy | 11 (9%) |
| Polycystic kidney disease | 3 (2.5%) |
| Chronic tubulointerstitial nephropathy | 3 (2.5%) |
| Other | 17 (14%) |
| Unknown | 32 (26.4%) |
| **Access type** |  |
| Arteriovenous fistula | 70 (57.9%) |
| Cuffed CVC | 16 (13.2%) |
| Uncuffed CVC | 25 (20.6%) |
| ^†^Unknown | 10 (8.3%) |
| **Duration of** ^‡^**ESKD (years)** | 3 ± 3.5 |

*Values are given as mean, +/- SD or N (%)

†Unclassified hemodialysis vascular access

‡End-stage kidney disease

§CVC central venous catheter

**Additional file 1: Table S2. ICD-10 codes used to define the etiology of kidney disease of the subset “other”**

| **Diagnosis** | **Code^*^ (ICD-10)** | **(N)** |
| --- | --- | --- |
|  |  |  |
| Malignant neoplasm of urinary organ - unspecified | DC689 | 3 |
|  |  |  |
| Amyloidosis | DE859 | 1 |
|  |  |  |
| Hypersensitivity angiitis | DM310 | 2 |
|  |  |  |
| Wegener’s granulomatosis | DM313 | 2 |
|  |  |  |
| Necrotizing vasculitis | DM319 | 1 |
|  |  |  |
| Recurrent and persistent haematuria – unspecified | DN028 | 1 |
|  |  |  |
| Renal tubulo-interstitial disease – unspecified | DN159 | 3 |
|  |  |  |
| Ischemia and infarction of kidney | DN280 | 1 |
|  |  |  |
| Neuromuscular dysfunction of bladder – unspecified | DN319 | 2 |
|  |  |  |
| Congenital hydronephrosis | DQ620 | 1 |
|  |  |  |
| Total |  | 17 |

Code* The Danish National Registry on Regular Dialysis and Transplantation

ICD-10 10^th^ revision of International Classification of Diseases

**Additional file 1: Table S3. International Classification of Diseases 8 and 10 codes used to define comorbidity and outcome in the study population**

| **Comorbidity** | **Diagnosis code** |
| --- | --- |
| Ischemic heart disease | ICD-8 411-414 |
|  | ICD-10 DI20, DI23-DI25 |
|  |  |
| Myocardial infarction | ICD-8 410 |
|  | ICD-10 DI21-DI22 |
|  |  |
| Heart failure | ICD-8 425, 514, 4270-4271 |
|  | ICD-10 DI42, DI110, DI50, J81 |
|  |  |
| Atrial flutter | ICD-8 42793-42794 |
|  | ICD-10 DI48 |
|  |  |
| Cardiac arrythmia | ICD-8 42721-42723, |
|  | ICD-10 DI44 |
|  |  |
| Diabetes mellitus | ICD-8 250 |
|  | ICD-10 E10-E14 |
|  |  |
| Chronic obstructive lung disease | ICD-8 490-492 |
|  | ICD-10 J42, J44 |
|  |  |
| Peripheral vascular disease | ICD-8 440, 443 |
|  | ICD-10 DI70, DI74 |
|  |  |
| Aortic valve disease | ICD-10 DI35 |
|  |  |
| Mitral valve disease | ICD-10 DI34 |
| **Outcome: Death** | **Diagnosis code** |
| Cardiovascular | ICD-10 DI109, DI21-DI22, DI251, DI259, DI33, DI34, DI35, DI38, DI48, DI429, DI469, DI461, DI490, DI509, DI519, DR570, DI61-DI64 |
| Non-cardiovascular | ICD-10 DA410, DA412, DA419, DE108, DE148, DJ960, DN18, DR029, DR092, DR99 |

ICD-8 8^th^ revision of International Classification of Diseases

ICD-10 10^th^ revision of International Classification of Diseases
